# Are the anti-arrhythmic effects of omega-3 fatty acids due to modulation of myocardial calcium handling?

**DOI:** 10.3389/fphys.2012.00373

**Published:** 2012-10-01

**Authors:** Rajiv Sankaranarayanan, Luigi Venetucci

**Affiliations:** ^1^Cardiovascular Research Group, University of ManchesterManchester, UK; ^2^Manchester Royal Infirmary, Manchester Heart CentreManchester, UK

**Keywords:** omega-3, fatty acids, anti-arrhythmics, calcium, fish oils

## Abstract

Both animal and clinical studies have demonstrated that omega-3 fatty acids have anti-arrhythmic properties. It has been suggested that these anti-arrhythmic effects are due to modulation of the activity of various myocardial calcium handling proteins such as ryanodine receptor (RyR), L-type calcium current and sodium/calcium exchanger. In this article, we review all the data available on the effects of omega-3 fatty acids on ventricular myocardial calcium handling. In addition we highlight some unanswered questions and discuss possible therapeutic benefits of omega-3 fatty acids.

## Introduction

Omega-3 poly-unsaturated fatty acids (PUFA) have generated considerable interest as well as controversy regarding their role as anti-arrhythmic agents. A number of observational studies have shown that consumption of fish leads to a reduction in incidence of sudden deaths (Burr et al., 1989; Siscovick et al., 1995). The GISSI–Prevenzione trial, an open-label design trial found that 1 g/day of n-3 PUFA [(containing 289 mg of Eicosapentaenoic acid (EPA) and 577 mg Docosahexaenoic acid (DHA)] led to a 20% Relative Risk Reduction (RRR, 95% CI 6–23%) in total mortality after only 3 months of treatment without a significant change in myocardial infarction or stroke (1999). This suggested that the beneficial effects of PUFA are mainly related to an anti-arrhythmic action that prevents life-threatening arrhythmias. This generated a great deal of interest in the potential of PUFA as anti-arrhythmic agents and 3 prominent randomized, double blind, placebo controlled trials have been conducted (Leaf et al., 2005; Raitt et al., 2005; Brouwer et al., 2006). These trials analyzed the effects of PUFA on time to appropriate therapy for ventricular arrhythmias in ICD patients. They failed to demonstrate a convincing anti-arrhythmic effect and ignited the debate on whether PUFA have anti-arrhythmic properties. It has been suggested that the lack of efficacy of PUFA in patients with ICDs is due to the fact that this population contains patients with various arrhythmia mechanisms while the main anti-arrhythmic actions of PUFA are due to modulation of calcium handling leading to prevention of delayed after-depolarization (DAD) and triggered activity (TA) (Den Ruijter and Coronel, 2009). PUFA have also been demonstrated to diversely modulate a variety of cardiac ion channels (inhibit sodium, calcium and potassium currents other than I_KS_ and I_K1_) as reviewed in detail in (Moreno et al., 2012) and hence the electrophysiological effects depend on the underlying cardiac pathology, setting of the arrhythmia and the method of application of PUFA. In this article we review the experimental evidence supporting the notion that the anti-arrhythmic actions of PUFA are due to modulation of myocardial calcium handling as well as prevention of DADs and TA. Whilst a variety of studies have also failed to reach a consensus on the effects of PUFA on the atrial myocardium, this review shall only focus on the effects of PUFA on calcium handling in the ventricular myocardium. To introduce the subject, we rapidly summarize normal myocardial calcium handling and the alteration in calcium handling that lead to DADs and TA.

## Myocardial calcium handling

**Figure 1:**
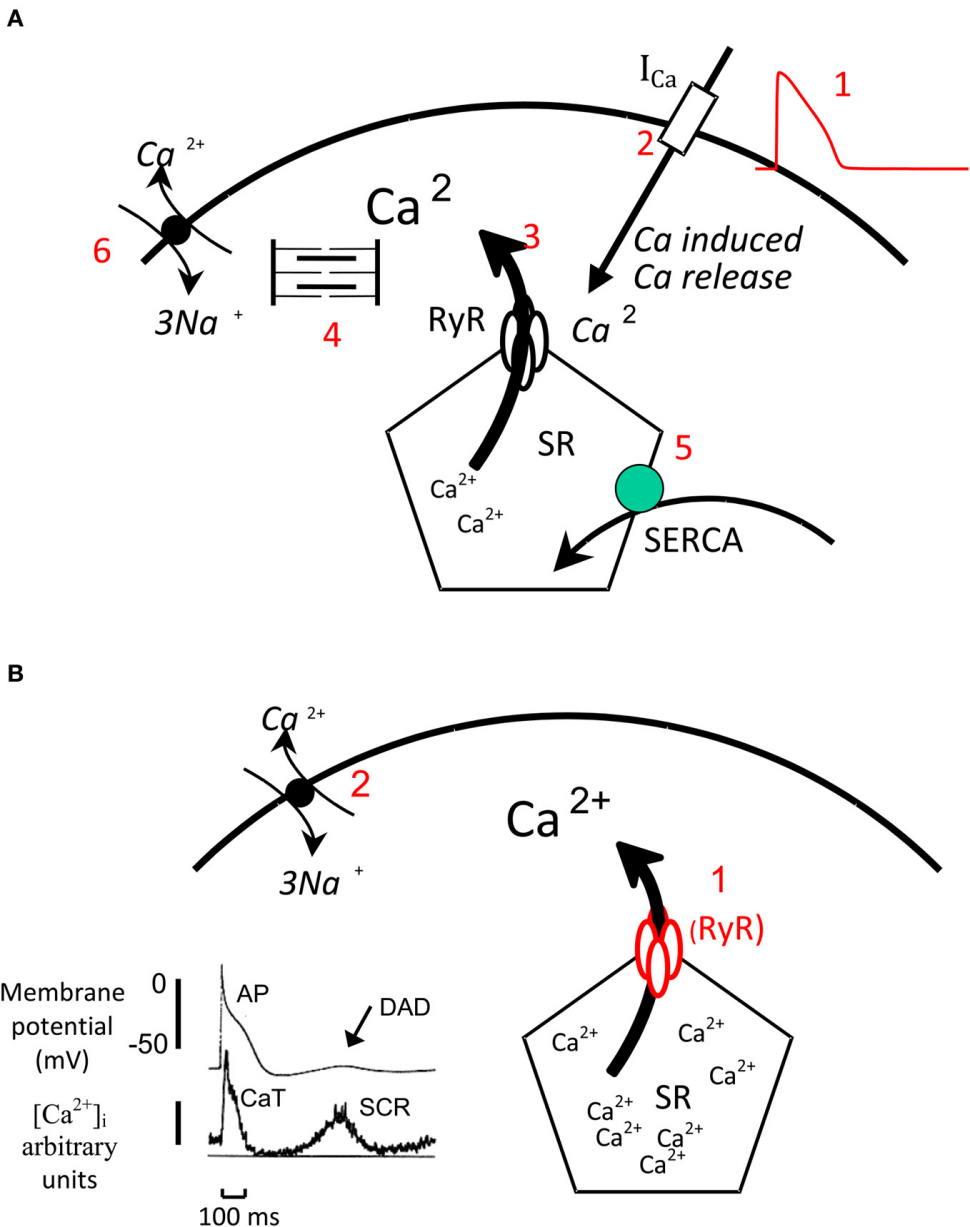
**Myocardial Calcium handling. (A)** Normal Ca handling: The AP (1) activates the L-type calcium channels, the influx of a small amount of calcium via these channels (2) activates the RyR and triggers the release of a greater amount of calcium from the SR in to the cytosol (3). Calcium activates the myofilaments that generate contraction. During relaxation calcium is rapidly removed from the cytosol by SERCA (5) that pumps calcium back into the SR and by NCX that couples the efflux of 1 Ca^2+^ (two positive charges) to the influx of 3 Na^+^ (three positive charges) and generates an inward current. **(B)** Generation of SCR and DADs: When intra SR calcium concentration is very high the SR can release calcium (1) independently from an AP this process is called spontaneous calcium release (SCR). This calcium activates the NCX (2) that generates an inward current and produces a delayed after—depolarization (DAD). The record on the left shows simultaneous recording of membrane potential and cytosolic calcium levels. The AP is triggers the calcium transient (CaT) while during diastole a SCR triggers a DAD.

In cardiac muscle, cytosolic calcium levels control the level of activation of the contractile proteins, the myofilaments and therefore the onset duration and intensity of contraction. The electrical activation with the spreading of the action potential (AP) throughout the heart initiates contraction by causing a transient increase in cytosolic calcium concentration, the systolic calcium transient (Bers, 2002). The AP generates the systolic calcium transient via a process called calcium-induced calcium release. During this process, the influx of a small amount of calcium via the sarcolemmal L-type calcium channels that are activated by the AP, activates the sarcoplasmic reticulum (SR) calcium release channel, the ryanodine receptor (RyR), and triggers the release of calcium from the SR. Relaxation occurs after 100–200 ms when calcium gradually decays back to diastolic levels. This decline in cytosolic calcium concentration is due to termination of calcium release from the SR (inactivation of RyR) and rapid removal of calcium from the cytosol. Two systems are responsible for calcium removal from the SR—the sarcoplasmic reticulum calcium ATPase (SERCA) that uses ATP as energy to pump calcium back into the SR and the Na^+^/Ca^2+^ exchanger (NCX) that exchanges the efflux of one Ca^2+^ (2 positive charges) with the influx of three Na^+^ (3 positive charges). The activation of NCX leads to a net influx of a positive charge and therefore a net inward current. The above process is shown in Figure 1A.

### Modulation of Ca handling

The amplitude of the calcium transient determines the level of activation of the myofilaments and therefore the intensity of contraction and it is finely modulated. The two main factors that determine the amount of calcium released from the SR and the amplitude of the calcium transient are: the amplitude of the L-type calcium current (Trafford et al., 2001) and the calcium concentration in the SR (Shannon et al., 2000). The SR Ca concentration can be increased by stimulation of SERCA activity and prolongation of the duration of the AP and decreased by stimulation of NCX activity.

*In vivo* the main modulator of the calcium transient amplitude is β adrenergic stimulation that via cAMP-mediated activation of protein kinase A stimulates both the L-type calcium channel and SERCA. This leads to an increase in L-type calcium current and SR Ca content and therefore a substantial increase in calcium transient amplitude (Hussain and Orchard, 1997).

### Arrhythmias related to Ca handling

Release of calcium from the SR can also occur independently from an AP during diastole (Venetucci et al., 2008). This process is called spontaneous calcium release (SCR) and occurs when the SR calcium concentration reaches a crucial threshold level (Diaz et al., 1997). In addition recently Belevych et al. (2012) elegantly demonstrated that soon after a systolic calcium transient the RyRs enter a refractory state and calcium wave occur only once the RyR have recovered from this refractory state even if the SR threshold is reached. Therefore the two conditions necessary for the generation of SCR are: (1) Increased SR Ca content up to the SR threshold for SCR (2) Recovery of RyR from refractory state. SCR activates the NCX that generates an inward current and a DAD. When a DAD reaches the threshold for activation of the Na channels, it initiates an AP which in turn causes TA and arrhythmias. The process of SCR and generation of DADs is shown in Figure 1B. SCR, DAD, and TA are responsible for the onset of arrhythmias in various clinical settings including digitalis-induced arrhythmias, some forms of heart failure and catecholaminergic polymorphic VT (a genetic arrhythmia syndrome caused by mutations of the RyR and CASQ2 genes). The scheme illustrated above suggests that there are two main therapeutic strategies that can be used to prevent the onset of calcium handling related arrhythmias: prevention of the onset of an SCR and prevention of triggering of an AP by a DAD. In view of the fact that the generation of DAD through SCR has been shown to be dependent on threshold SR calcium content (Diaz et al., 1997; Trafford et al., 2000; Jiang et al., 2005), the first strategy can be achieved by preventing the SR calcium content from reaching the threshold for SCR either by decreasing SR calcium content (Venetucci et al., 2007; Llach et al., 2011) or by raising SR threshold for SCR via inhibition of RyR (Venetucci et al., 2006; Maxwell et al., 2012). The second strategy can be achieved via inhibition of sodium channels that reduces the number of sodium channels ready to be activated when a DAD occurs and therefore reduces the likelihood that the DAD will initiate and AP and cause TA. Whilst this strategy can be beneficial in preventing arrhythmias caused by TA such as in heart failure, it could also be pro-arrhythmic during acute ischaemia by facilitating re-entry. In the following section, we will illustrate the experimental evidence that suggests that PUFA prevent SCR, DADs and TA via some of the mechanisms described.

## Effects of PUFA on myocardial calcium handling

The effects of PUFA on calcium handling by cardiac myocytes have been extensively studied and an interesting picture has emerged. The effects vary depending on the modality of administration; two different modalities of application have been used: (1) application of free unesterified PUFA in external bath solution, (2) chronic diet supplementation that leads to incorporation in cardiac membrane as esters. To simplify, we will describe these effects separately.

## Effects of free PUFA on myocardial calcium handling (also summarised in Table 1)

Application of free PUFA has profound effects on calcium handling. Macleod et al studied the effects of free PUFA on sodium and calcium currents and AP (Macleod et al., 1998). Both in rat as well as guinea pig isolated cardiac myocytes, free PUFA produced a dose-dependent reduction in sodium and calcium currents. The effects on AP duration were different in the two species. In rat ventricular myocytes, concentrations of EPA or DHA up to 7.5 μM caused AP prolongation. At concentrations above 10 μM AP shortening was observed. In guinea-pigs however AP shortening was observed at lower concentrations such as around 5 μM. In a second paper the same authors (Rodrigo et al., 1999) also demonstrated that PUFA at the same concentrations inhibit RyR. The inhibition of calcium current and the RyR and the shortening of the AP produce marked reduction in calcium transient amplitude and cell shortening. Stephen O'Neill's group studied in detail the effects of PUFA on Ca handling in rat ventricular myocytes (Negretti et al., 2000; O'Neill et al., 2002). They first studied the effects of EPA and DHA on RyR and confirmed that PUFA inhibit RyR and also increase the SR threshold for SCR (Negretti et al., 2000). In the same paper, they also demonstrated that free PUFA have no effects on NCX. In a follow-on paper (Szentandrássy et al., 2007), they also analyzed the effects of EPA on L-type calcium current, SERCA function and calcium transient. As previously described by Rodrigo et al. EPA reduces calcium current amplitude (Rodrigo et al., 1999). Interestingly EPA also increased SERCA activity by promoting phosphorylation of phospholamban. The characterization of the calcium transient demonstrated that 5 μM EPA reduced calcium transient if the myocytes were stimulated using voltage-clamp and increased it if the cell was stimulated using current-clamp. During current-clamp the cells are allowed to express their AP and the application of EPA (via inhibition of the transient outward current) produces a substantial prolongation of the AP. This increases SR calcium content substantially and therefore increases calcium transient amplitude despite inhibition of the L-type calcium current. During voltage clamp the cell is not allowed to express its AP. The membrane potential is controlled and the cell is stimulated using a 100 ms membrane potential step. The application of EPA does not produce any change in membrane potential and therefore does not produce a significant increase in SR Ca content. EPA still reduces calcium current amplitude and this reduction cause a significant reduction in calcium transient amplitude.

**Table 1:** **Summarising effects of free PUFA on myocardial calcium handling**.

**Authors**	**Species studied**	**Findings**
Macleod et al., 1998	Rat and guinea pig isolated cardiac myocytes	1. Dose dependant reduction in sodium and calcium currents
		2. Rat - or DHA up to 7.5 μM caused AP prolongation Guinea pig—AP shortening was observed already at lower concentrations such as around 5 μM
Rodrigo et al., 1999	Rat and guinea pig isolated cardiac myocytes	Inhibition of calcium current and the RyR and the shortening of the AP produce marked reduction in calcium transient amplitude and cell shortening
Negretti et al., 2000 and O'Neill et al., 2002	Rat ventricular myocytes	1. PUFA inhibit RyR and also increase the SR threshold for SCR
		2. Free PUFA have no effects on NCX
Szentandrássy et al., 2007	Rat ventricular myocytes	1. EPA reduces calcium current amplitude
		2. EPA also increased SERCA activity by promoting phosphorylation of phospholamban
		3. The characterization of the calcium transient demonstrated that 5 μM EPA reduced calcium transient if the myocytes were stimulated using voltage-clamp and increased it if the cell was stimulated using current-clamp
**EFFECTS ON ADRENERGIC STIMULATION**		
Kang and Leaf, 1995	Rat neonatal myocytes	Application of free PUFA to spontaneously beating rat neonatal myocytes attenuated the response to isoprenaline, reduced the increase in beating frequency produced by isoprenaline and prevented the onset of contracture
Den Ruijter et al., 2008	Rabbit and human myocytes	In rabbit myocytes, free PUFA reduced amplitude of the calcium transient and attenuated the increase in calcium transient amplitude produced by noradrenaline. In addition, in the presence of PUFA noradrenaline did not prolong the AP and failed to induce EADs and DADs.
		Similar effects were detected during experiments on human cardiac myocytes derived from severe congestive cardiac failure hearts explanted during cardiac transplantation.
Szentandrássy et al., 2007	Rat ventricular myocytes	PUFA reduce cAMP levels but directly stimulate PKA

**Figure 2:**
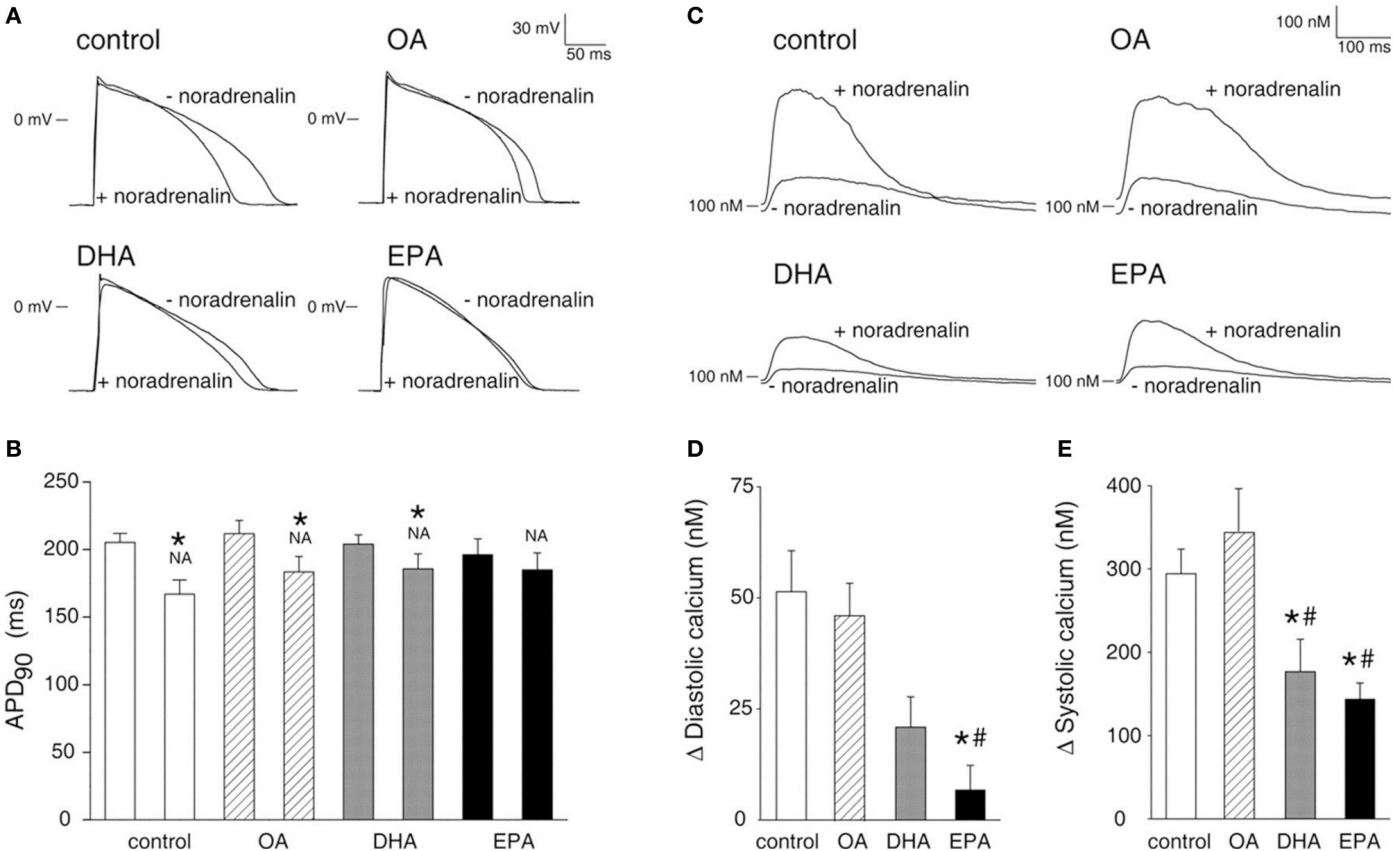
**PUFA reduce the sensitivity to noradrenalin. (A)** Effects of free Oleic acid (OA), free EPA and free DHA on AP duration before and after application of noradrenalin. **(B)** Mean data for AP duration. ^*^ *P* < 0.05 indicates statistical differences with or without noradrenalin. **(C)** Effects of free OA free EPA and free DHA on calcium transient before and after application of noradrenalin. **(D)** Mean data for diastolic calcium levels. ^*^P < 0.05 indicates statistical differences between DHA or EPA and control. **(E)** Mean data for peak systolic calcium levels. # *P* < 0.05 indicates statistical differences between DHA or EPA and OA. Picture reproduced with permission from Den Ruijter et al. Circulation (2008) 117, 536–544.

Free PUFA have also been shown to reduce the response to adrenergic stimulation. In 1995 Kang and Leaf (1995) demonstrated that the application of free PUFA to spontaneously beating rat neonatal myocytes attenuated the response to isoprenaline. PUFA reduced the increase in beating frequency produced by isoprenaline and prevented the onset of contracture. More recently, Den Ruijter et al. (2008) characterized the effects of PUFA on the adrenergic response in rabbit and human myocytes (shown in Figure 2). In rabbit myocytes, free PUFA reduced the amplitude of the calcium transient and attenuated the increase in calcium transient amplitude produced by noradrenaline. In addition, in the presence of PUFA noradrenaline did not prolong the AP and failed to induce EADs and DADs. Similar effects were detected during experiments on human cardiac myocytes derived from severe congestive cardiac failure hearts explanted during cardiac transplantation. The mechanisms responsible for the attenuation in adrenergic response are not fully understood. Szentandrássy et al. (2007) demonstrated that PUFA reduce cAMP levels but directly stimulate PKA. It is conceivable that during adrenergic stimulation PUFA significantly attenuate the increase in cAMP levels and therefore attenuate the response to adrenergic stimulation. In summary, these studies have consistently demonstrated that free PUFA *in vitro* exert profound effects on calcium handling at baseline and after adrenergic stimulation. These effects involve several components of the calcium handling system and produce marked reduction in calcium transient amplitude. These studies also suggest that free PUFA prevent DADs *in vitro* both by raising SR threshold for calcium waves (through inhibition of RyR) and by reducing the SR calcium content both before and after adrenergic stimulation. In addition, they inhibit sodium current and therefore prevent TA. It is unclear whether these effects also occur *in vivo* (see later).

## Effects of membrane incorporated PUFA

As mentioned above, dietary supplementation with EPA and DHA leads to their incorporation in the cardiac membrane phospholipids. Several studies have investigated the effects of incorporated PUFA but have reached conflicting reports. To simplify the subject we illustrate separately the effects of incorporated PUFA on calcium handling at baseline and following adrenergic stimulation.

### Effects on calcium handling at baseline

In 2001 Leifert et al. (2001) gave a DHA and EPA enriched diet to adult rats for 3 weeks. This diet increased the levels of incorporation of DHA (from 6 to 20% of total phospholipids) and EPA (from 0 to 3.2%). The incorporated PUFA did not affect calcium transient amplitude but decreased its rate of decay. In addition, incorporation of PUFA did not affect SR Ca content. The authors suggested that the reduction in calcium transient rate of decay was due to reduced NCX-mediated removal in the PUFA group. It is likely that the effects are more complex because isolated reduction of NCX function would have also increased SR calcium content. The Coronel group studied the effects of a PUFA-enriched diet on pig ventricular myocytes. The diets were given for 8 weeks and produced a significant increase in the levels of incorporated DHA (from not detectable to around 7%) and EPA (from not detectable to around 15%). The most striking effect of incorporated PUFA was reduction in AP duration which was more prominent at slow frequencies and was mainly due to increase in two repolarizing currents: the slow component of the delayed rectifier current and inward rectifier K current (Den Ruijter et al., 2006; Verkerk et al., 2006). The analysis of calcium handling showed that the incorporated PUFA did not produce a significant effect on calcium transient amplitude but accelerated its rate of decay. SR calcium content was not affected but both L-type calcium current amplitude and NCX function were significantly reduced. The acceleration of the calcium transient rate of decay is probably explained by the shorter AP duration, but what is surprising is the fact that despite reduction in L-type calcium current amplitude, the calcium transient amplitude itself was not affected. Recently Billman et al. (2010) assessed the effects of DHA and EPA supplements (Omacor 1–2 or 4 tablets a day) on contractility and calcium transient in mixed breed dogs. The tablets were given for 3 months. The supplements produced a significant increase in EPA and DHA incorporation levels in cardiac membranes. However, the increased incorporation levels did not produce any significant effect on cardiac contractility, calcium transient amplitude and on L-type calcium current amplitude.

### Effects on response to adrenergic stimulation

Leifert et al. (2001) reported that incorporation of DHA and EPA in membranes of rat ventricular myocytes reduced the incidence of calcium waves and DADs during challenge with the β agonist isoproterenol. From the data illustrated by the authors, it is difficult to understand the mechanisms responsible for these protective effects. The authors also showed that the incorporation of DHA and EPA does not affect the increase in calcium transient amplitude produced by isoproterenol. Similarly to what described at baseline there was a reduction in the rate of decay of the calcium transient that was significant only at 0.2 Hz. From these data is difficult to establish how incorporated PUFA reduced the incidence of calcium waves. It is not clear whether PUFA affected SR threshold for calcium waves and/or SR calcium content. Similar to what was previously described by Leifert et al. in rat cardiac myocytes, the Coronel group demonstrated that incorporation of DHA and EPA in the membrane of pig ventricular myocytes reduced the incidence of calcium waves and DADs during challenge with norepinephrine (Berecki et al., 2007). They investigated in detail the mechanism responsible for these effects and demonstrated that membrane incorporation of PUFA reduces the response to norepinephrine. In particular, incorporated PUFA blunted the increase in SR calcium content produced by norepinephrine and therefore attenuated the increase in calcium transient produced by norepinephrine. Incorporated PUFA also prevented the prolongation of AP potential produced by norepinephrine and it is unclear whether the blunting in the increase of SR calcium content is simply due to the effects on AP duration or also involves blunting of the stimulation of SERCA activity. In a recent paper, Billman et al. showed that incorporation of DHA and EPA in cardiac membranes of dogs did not affect the response to isoproterenol. The authors however did not investigate whether incorporated PUFA prevented the onset of calcium waves and DADs. However the finding that they do not attenuate response to isoproterenol would suggest that in these experiments they would not prevent DADs. An important issue that was not investigated by all these studies is whether incorporated PUFA have any effect on RyR and affect the threshold for SCR. Some studies inferred that because calcium transient amplitude was not affected there was no effect on RyR.

In summary, the studies that have investigated the effects of incorporated PUFA on Ca handling have reached conflicting conclusions. The cause of these differences remains unclear. Billman et al. have suggested that they may be related to the different species utilized in the studies. More specifically pigs (utilized by the Coronel Group) have a calcium-dependent transient outward current that is absent in dog and human myocytes. This could cause different effects of incorporated PUFA on AP duration and therefore SR calcium content before and after adrenergic stimulation. However this does not explain the differences in the effects of incorporated PUFA on the L-type Ca current. In addition, the pig study that documented attenuated adrenergic response used norepinephrine (β1 and α1 agonist) to produce adrenergic stimulation while the other two studies that documented limited effects utilized isoproterenol (β1 and β2 agonist). This raises the possibility that stimulation with norepinephrine is more susceptible to modulation by incorporated PUFA. This is a possibility that needs to be investigated.

## Combined effects of free and membrane incorporated PUFA

One issue that has not been addressed by the studies performed is whether incorporation of PUFA in cardiac membrane affects the response to free PUFA. The issue is particularly important because diet supplementation leads both to membrane incorporation and to an increase in free circulating PUFA. Only one study has tried to address this issue (Den Ruijter et al., 2010) and has demonstrated that incorporation of PUFA in the membrane of rabbit myocytes shortens AP but prevents any further shortening when free PUFA are applied. Unfortunately this study has not determined whether the effects of free PUFA on calcium handling (inhibition of RyR and L-type calcium current) are affected by incorporated PUFA.

### Do the effects of PUFA on calcium handling detected *In vitro* occur *In vivo* as well?

The large amount of experimental evidence gained with *in vitro* experiments has not been supported by *in vivo* experiments. Several studies have demonstrated that *in vivo* both infusion of PUFA (free PUFA) (Billman et al., 1999) and chronic dietary supplementation (that leads to membrane incorporation and increased circulating free PUFA) protect from ischaemia-reperfusion related arrhythmias (McLennan et al., 1988; London et al., 2007). However a recent study evaluating the effects of dietary n-3 PUFA on susceptibility to post-myocardial infarction ventricular fibrillation in dogs, showed that despite significant increases in circulating as well as left ventricular PUFA levels, PUFA not only failed to prevent ischaemia-induced VF. Contrary to expectations dietary PUFA exerted pro-arrhythmic effects facilitating the onset of VF both in non-infarcted animals and in low-risk post-MI dogs that did not have VF prior to initiation of PUFA diet (Billman et al., 2012). These conflicting results highlight the fact that the pathogenesis of ischaemia reperfusion related arrhythmias is complex and certainly does not involve just alterations in calcium handling (Janse and Wit, 1989). Therefore this experimental evidence cannot be used as proof that PUFA modulate calcium handling *in vivo*. The evidence that PUFA *in vivo* modulate myocardial calcium handling is limited. This question could be answered by studies on cardiac contractility and on calcium handling related arrhythmias. To date no study has assessed the effects of acute PUFA administration on cardiac function and the two studies that have assessed the effects of PUFA diet supplementation on cardiac function have not confirmed the *in vitro* findings. In 1992, McLennan et al. (1992) demonstrated that dietary supplementation with PUFA in marmosets increased ejection fraction by enhancing LV filling. Recently Billman et al. (2010) studied the effects of PUFA supplementation on LV function (assessed by echo) and demonstrated that despite significant increases in the incorporation of DHA and EPA in the cardiac membrane (and probably in circulating free PUFA) there was no change in LV function. Interestingly these results are very different from what one would expect on the basis of *in vitro* studies that point more towards a reduction in calcium transient amplitude, cardiac contractility and LV function. These data suggest that *in vivo* there are complex modulating factors that mitigate or abolish the effects of PUFA observed *in vitro*. The evidence that PUFA prevent arrhythmias which are exclusively due to abnormalities in calcium handling, calcium waves and DADs (such as catecholaminergic and digoxin-related arrhythmias) is also limited. Only one study (Gudbjarnason et al., 1989) demonstrated that in rats a PUFA diet prevented the onset of VF following isoproterenol infusion. There is clearly a need for more targeted studies that specifically assess the anti-arrhythmic potential of PUFA in arrhythmia syndromes caused exclusively by abnormalities in calcium handling. To this purpose, a study in animal models and or patients with CPVT would address this point.

## Conclusions

A large body of evidence gained from cellular experiments supports the idea that PUFA modulate myocardial calcium handling and exert anti-arrhythmic effect by preventing SCR and DADs. This large body of *in vitro* evidence still awaits confirmation by *in vivo* animal studies and clinical studies. Over the next decade targeted studies will tell us whether all these *in vitro* findings also occur *in vivo* and whether PUFA are a treatment strategy for calcium handling related arrhythmias.

### Funding

Dr. Sankaranarayanan's research is funded by a grant from the British Heart Foundation (Grant Reference FS/11/15/28693). Dr. Venetucci's research is funded by a grant from the British Heart Foundation (Grant Reference FS/10/63/28374).

### Conflict of interest statement

The authors declare that the research was conducted in the absence of any commercial or financial relationships that could be construed as a potential conflict of interest.

## Abbreviations

PUFA: Poly-Unsaturated Fatty Acids
EPA: Eicosapentaenoic acid
DHA: Docosahexaenoic acid
ICD: Internal cardioverter-defibrillator
DAD: delayed afer-depolarization
TA: triggered activity
AP: action potential
SR: sarcoplasmic reticulum
RyR: ryanodine receptor
SERCA: Sarcoplasmic reticulum Ca ATPase
ATP: adenosine triphosphate
NCX: Na^+^/Ca^2+^ exchanger
SCR: spontaneous calcium release
VT: ventricular tachycardia
EAD: early after-depolarization
VF: ventricular fibrillation.
